# Supplementary material for: ProteinShader: illustrative rendering of macromolecules
Source: BMC Struct Biol. 2009 Mar 30;9:19. doi: 10.1186/1472-6807-9-19 (PMC2672931; doi:10.1186/1472-6807-9-19)
Supplement: Additional file 1 — ProteinShader program without source code. This compressed file contains the complete ProteinShader program including associated libraries, but no source code. A README.txt file gives an overview of the ProteinShader distribution, and the index.html file in the help subdirectory has directions on getting started with the program as well as a set of tutorials. [file 1472-6807-9-19-S1.zip › ProteinShader-beta-0_9_4-binary/help/api/org/proteinshader/gui/enums/package-use.html]

Uses of Package org.proteinshader.gui.enums (ProteinShader API)


|  |  |  |
| --- | --- | --- |
| **Overview** \| **Package** \| Class \| **Use** \| **Tree** \| **Deprecated** \| **Index** \| **Help** | |  |
| PREV   NEXT | **FRAMES**    **NO FRAMES**     **All Classes** |  |


---


## **Uses of Package org.proteinshader.gui.enums**

| Packages that use org.proteinshader.gui.enums | |
| --- | --- |
| **org.proteinshader.graphics.adapter** | Holds the StructureToGraphics class, which is used to manage the use of the drawing classes and OpenGL display lists. |
| **org.proteinshader.graphics.displaylists** | Holds the classes needed to manage OpenGL display lists, which are used to cache reusable geometry for spheres, cylinders, ribbon segments, and tube segments. |
| **org.proteinshader.gui** | Holds all of the Swing GUI components and their associated listeners, including class Renderer, which is registered as a listener for the GLCanvas object that is used a drawing surface. |
| **org.proteinshader.gui.components** | Holds the gui components (JPanels and JMenus) needed by class ProteinShaderGUI. |
| **org.proteinshader.gui.components.controlpanel** | Holds the JPanels used in the right-side retractable control panel of the GUI. |
| **org.proteinshader.gui.components.menubar** | Holds the JMenus used in menu bar above the canvas. |
| **org.proteinshader.gui.enums** | Holds the enums needed by org.proteinshader.gui and its subpackages. |
| **org.proteinshader.gui.exceptions** | Holds the exception classes needed by org.proteinshader.gui and its subpackages. |
| **org.proteinshader.gui.utils** | Holds minor utility classes needed by the Renderer. |
| **org.proteinshader.gui.viewing** | Holds important helper classes that the Renderer uses to control the view: Camera, Rotation, Lighting, *etc*. |

Classes in org.proteinshader.gui.enums used by org.proteinshader.graphics.adapter

****StyleEnum****
  
          Provides an enumeration to specify what style should be used to display a
Structure: Cartoon (Tubes and Ribbons) or Atom (Spheres and Cylinders).

Classes in org.proteinshader.gui.enums used by org.proteinshader.graphics.displaylists

****StyleEnum****
  
          Provides an enumeration to specify what style should be used to display a
Structure: Cartoon (Tubes and Ribbons) or Atom (Spheres and Cylinders).

Classes in org.proteinshader.gui.enums used by org.proteinshader.gui

****ImageFormatEnum****
  
          Provides an enumeration of the image file formats.

****JitterEnum****
  
          Holds the xy-coordinates needed for jittering a scene to accomplish
antialiasing (the smoothing of jagged edge lines).

****OrientationEnum****
  
          Provides a general orientation enumeration.

****StyleEnum****
  
          Provides an enumeration to specify what style should be used to display a
Structure: Cartoon (Tubes and Ribbons) or Atom (Spheres and Cylinders).

Classes in org.proteinshader.gui.enums used by org.proteinshader.gui.components

****OrientationEnum****
  
          Provides a general orientation enumeration.

Classes in org.proteinshader.gui.enums used by org.proteinshader.gui.components.controlpanel

****JitterEnum****
  
          Holds the xy-coordinates needed for jittering a scene to accomplish
antialiasing (the smoothing of jagged edge lines).

****RadioButtonEnum****
  
          Provides an enumeration of the types of radio buttons in the
RadioPanel class.

****StyleEnum****
  
          Provides an enumeration to specify what style should be used to display a
Structure: Cartoon (Tubes and Ribbons) or Atom (Spheres and Cylinders).

Classes in org.proteinshader.gui.enums used by org.proteinshader.gui.components.menubar

****StyleEnum****
  
          Provides an enumeration to specify what style should be used to display a
Structure: Cartoon (Tubes and Ribbons) or Atom (Spheres and Cylinders).

Classes in org.proteinshader.gui.enums used by org.proteinshader.gui.enums

****ImageFormatEnum****
  
          Provides an enumeration of the image file formats.

****JitterEnum****
  
          Holds the xy-coordinates needed for jittering a scene to accomplish
antialiasing (the smoothing of jagged edge lines).

****OrientationEnum****
  
          Provides a general orientation enumeration.

****RadioButtonEnum****
  
          Provides an enumeration of the types of radio buttons in the
RadioPanel class.

****StyleEnum****
  
          Provides an enumeration to specify what style should be used to display a
Structure: Cartoon (Tubes and Ribbons) or Atom (Spheres and Cylinders).

Classes in org.proteinshader.gui.enums used by org.proteinshader.gui.exceptions

****ImageFormatEnum****
  
          Provides an enumeration of the image file formats.

Classes in org.proteinshader.gui.enums used by org.proteinshader.gui.utils

****ImageFormatEnum****
  
          Provides an enumeration of the image file formats.

Classes in org.proteinshader.gui.enums used by org.proteinshader.gui.viewing

****ImageFormatEnum****
  
          Provides an enumeration of the image file formats.

****OrientationEnum****
  
          Provides a general orientation enumeration.

---


|  |  |  |
| --- | --- | --- |
| **Overview** \| **Package** \| Class \| **Use** \| **Tree** \| **Deprecated** \| **Index** \| **Help** | |  |
| PREV   NEXT | **FRAMES**    **NO FRAMES**     **All Classes** |  |


---

# *Copyright © 2007-2008*
